# Aortitis: An Unusual Inflammatory Complication of Systemic Lupus Erythematosus

**DOI:** 10.7759/cureus.18028

**Published:** 2021-09-16

**Authors:** Juan Camilo Santacruz, John Dario Londoño, Uriel Panqueva, Francy Cuervo

**Affiliations:** 1 Spondyloarthropathies Research Group, Universidad de la Sabana, Chía, COL; 2 Rheumatology Department, Fundación Cardioinfantil, Bogotá, COL

**Keywords:** lymphoplasmacytic infiltrate, glucocorticoids, refractory antiphospholipid syndrome, systemic lupus erythematosus, aortitis

## Abstract

Systemic lupus erythematosus (SLE) is a chronic autoimmune disease that can involve any organ system. Vasculitides are classified according to the predominant vessel involved such as large vessel, medium vessel, or small vessel vasculitis. Of these, Takayasu arteritis, Behcet's disease, relapsing polychondritis, and immunoglobulin G4 (IgG4)-related disease predominantly involve large vessels. The most common form of vasculitis seen in SLE is small vessel vasculitis. Aortitis in SLE is an extremely rare complication. This is a case report of a 21-year-old female patient with a history of SLE with overlap syndrome of primary biliary cirrhosis and autoimmune hepatitis associated with antiphospholipid syndrome (APS), who presented with a one-week history of left-back burning lumbar pain, radiating to the flank, which increased with changes in position associated with intermittent claudication. In the angiography images and the positron emission tomography (PET) scan, a hypometabolic left para-aortic oval image was noted, corresponding to the presence of a contained hematoma in an abdominal aorta rupture. Later, she underwent vascular surgery and hemodynamics, performing thoracoabdominal aortic reconstruction together with aortorenal bypass and left nephrectomy. Pathology fundings of the left kidney correspond with class IV lupus nephritis, and the resection sample of the thoracoabdominal aneurysm showed a marked thinning and fragmentation of elastic fibers, areas of fibrosis of the wall with severe IgG4 negative lymphoplasmacytic infiltrate in the immunohistochemical study, establishing the diagnosis of aortitis.

## Introduction

The estimated prevalence of vasculitis in systemic lupus erythematosus (SLE) patients from large cohorts varies between 11% and 36% [1]. The clinical spectrum of vasculitis in the context of SLE is very broad due to the potential inflammatory involvement of either small, medium, or large vessels. Small-vessel involvement is the most common manifestation, often appearing as skin lesions. A few cases of aortitis, similar to those seen in Takayasu arteritis have been reported, however, its etiology is unclear. Treatment represents a great challenge for multiple specialties while endovascular interventions are generally required along with adjustments in immunosuppressive therapy associated with the correction of risk factors involved in the pathogenesis of arterial disease.

## Case presentation

**Figure 1:**
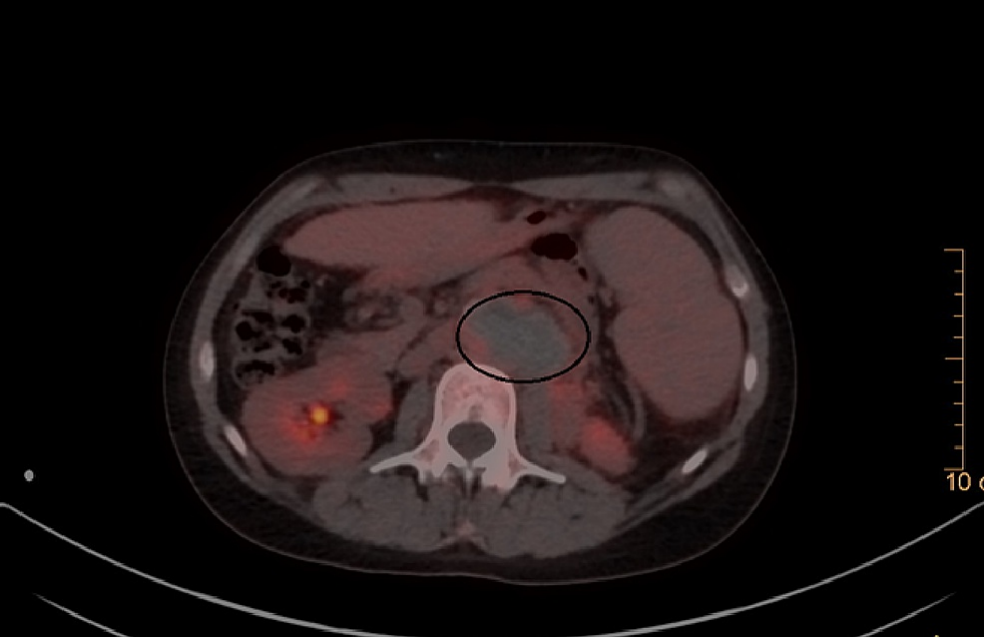
PET/CT with 18F-fluorodeoxyglucose (axial section) A left para-aortic oval image is described (highlighted by a black circle) with central hypometabolism and scarce peripheral metabolism, measuring approximately 55 * 33 mm in the axial plane, suggestive of a hematoma contained in the abdominal aorta. PET: positron emission tomography

**Figure 2:**
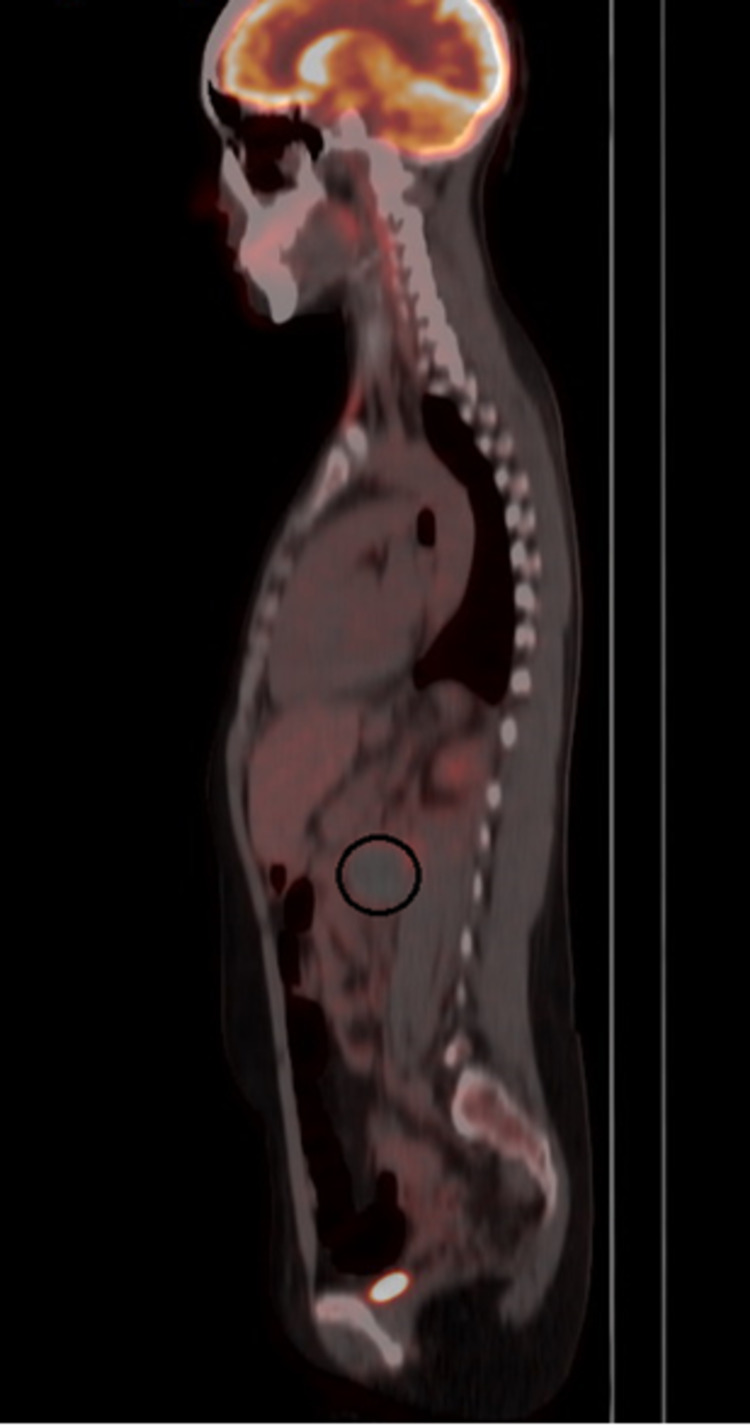
PET/CT with 18F-fluorodeoxyglucose (sagittal section) The left para-aortic oval image described in Figure 1 is described in a sagittal section highlighted with a black circle. PET: positron emission tomography

A 21-year-old female patient with a history of SLE with overlap syndrome of primary biliary cirrhosis and autoimmune hepatitis confirmed by an antinuclear antibody (ANA)-positive 1/640 reticular cytoplasmic pattern, hypocomplementemia, and anti-DNA at high titers associated with antiphospholipid syndrome (APS) with a thrombotic phenotype (previous history of infrarenal aortic thrombosis, cardiolipin immunoglobulin M (IgM) and B2 IgM glycoprotein in high titers) initially anticoagulated with warfarin with INR in therapeutic range since November 2020. The patient was treated with prednisolone at 1 mg/kg, chloroquine 250 mg per day, and azathioprine 50 mg every 12 hours. She initially presented with a history of one week of burning pain in the back of the lumbar region, radiating to the flank of the same side, which increased with changes in position associated with intermittent claudication. Abdominal ultrasound done as an outpatient (prior to the presentation) revealed left renal hypoplasia and splenomegaly. As an initial study, a renal artery doppler was performed showing post-stenotic left renal intraparenchymal arterial flow, which suggested the possibility of thrombosis. Subsequently, angiography showed an infrarenal aortic occlusion with distal reconstitution of the iliac arteries and a left para-aortic retroperitoneal lesion that suggested a lymph node conglomerate as the first possibility. Left renal atrophic changes were also identified with images suggestive of vascular infarcts. The possibility of an infectious process was not ruled out; consequently, a PET scan was indicated, identifying a hypometabolic left para-aortic oval image that corresponded to a hematoma with contained rupture of the abdominal aorta (Figures 1-2). 

**Figure 3:**
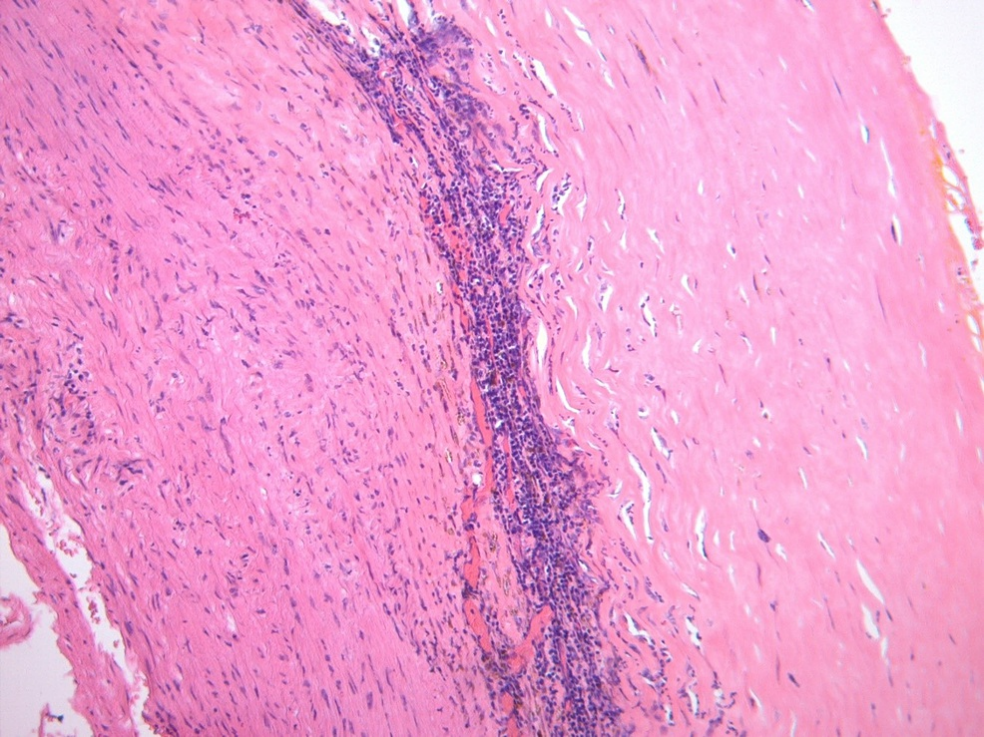
Microscopic description of the resected thoracoabdominal aneurysm Marked thinning and fragmentation of elastic fibers with areas of fibrosis of the wall is observed along with severe Ig4-negative lymphoplasmacytic infiltrate by immunohistochemical study. PAS, ZN, and Grocott stains did not show the presence of microorganisms.

No hypermetabolic alterations consistent with an active infectious process were observed. Vascular surgery and hemodynamics were consulted, and she underwent performing a thoracoabdominal aortic reconstruction together with an aortorenal bypass and left nephrectomy. Renal arteriography revealed a 99% subocclusive thrombotic lesion, for which balloon angioplasty was done along with the deployment of a stent to normalize the lumen of the vessel. The postoperative course was complicated by acute kidney injury (KDIGO-3) requiring temporary hemofiltration and right hemothorax requiring surgical drainage. The findings of the pathology of the left kidney corresponded to a class IV lupus nephritis and the resection sample of the thoracoabdominal aneurysm showed a marked thinning and fragmentation of elastic fibers, areas of fibrosis of the wall, with severe immunoglobulin G4 (IgG4)-negative lymphoplasmacytic infiltrate in the study of immunohistochemistry, establishing the diagnosis of aortitis (Figure 3).

The rate of erythrocyte sedimentation upon admission was 44 mm/h, the urinalysis showed proteinuria and hematuria that were interpreted to be expected due to the renal thrombotic event but given the findings of the pathology, it was concluded that they were attributed to the activity of the disease. Below is a table with the description of the most representative paraclinical studies upon admission and during hospitalization (Table 1).

**Table 1:** Laboratory parameters at admission, in the intensive care unit, and during the outpatient consultation ESR: erythrocyte sedimentation rate; ALT: alanine transaminase; AST: aspartate aminotransferase; ANAs: antinuclear antibodies; ENAs: extractable nuclear antigens; Ig: immunoglobulin; CRP: c-reactive protein; INR: international normalized ratio

Paraclinical studies	Upon admission	Stay in ICU	Outpatient
Hemoglobin (gr/dL)	12.7	8.04	14.3
Hematocrit (%)	36.8	23.3	43.5
Mean corpuscular volume (fL)	75.9	80.3	83.3
Total leukocyte count (/µl)	9660	11200	6611
Total lymphocyte count (/µl)	1370	2350	1830
Total platelet count (/µL)	181.000	141.000	329.000
Creatinine (mg/dL)	0.7	4.5	0.8
Ureic nitrogen (mg/dL)	5	31	22
Potassium (mEq/L)	3.7	5.9	4.7
Sodium (mEq/L)	136	134	138
ESR (mm/h)	44	18	7
ALT U/L	16	6	333
AST U/L	26	32	357
ANAs (titers)	Hep 2 positive 1/640	-	-
ENAs (U/mL)	Not known	-	-
Anti-DNA (IU/mL)	1/320	-	-
C3 (mg/dL)	0.6	-	-
C4 (mg/dL)	0.06	-	-
Cardiolipin IgG (U/ml)	18.8	-	-
Cardiolipin IgM (U/ml)	˃150	-	-
Lupus anticoagulant	Negative	-	-
CRP (mg/dL)	-	1.2	0.2
INR	3.03	1.3	1.29
Prothrombin time (sec)	44.9/14	16/14	18/14
Thromboplastin time (sec)	44.7/30	66/30	56.7/30
24-hour urine protein	268.4 mg / 24 hours	-	-
Uroanalysis	Hyaline cylinders	Hyaline cylinders	-

## Discussion

Vasculitides are classified according to the predominant vessel involved such as large vessel, medium vessel, or small vessel vasculitis. Of these, Takayasu arteritis, Behcet's disease, relapsing polychondritis, and IgG4-related disease predominantly involve large vessels [2]. The clinical spectrum of vasculitis in the setting of SLE is broad due to the potential for inflammatory involvement of vessels of all sizes. However, aortitis is an extremely rare complication, and without the intensification of immunosuppressive treatment and timely endovascular interventions, it can become a life-threatening condition. Additionally, very little is known about its pathophysiology and there are not any randomized controlled studies that dictate a clear guideline regarding its treatment. Described cases are very heterogeneous, and the vast majority have no histopathological confirmation. There has also been a relationship in the genesis of aortic aneurysms or dissection caused by long-term glucocorticoid treatment since its administration itself can induce atherosclerotic changes and contribute to the fragility of the tunica media causing its thinning. However, the patient was recently diagnosed in spite of a dose of prednisolone at 1 mg/kg for nine months, and the histopathological involvement supports a frank inflammatory involvement, unlike the non-inflammatory findings observed with the administration of glucocorticoids. Histopathology findings generally reveal vasculitic involvement of the vasa nervorum, diffuse lymphocytic infiltration, and necrosis of the elastic tunic without the presence of giant cells, granulomas, and neutrophils. Paradoxically, there are reports of cases that have achieved an adequate response with intermediate doses of prednisolone (0.5 mg/kg/day) with an improvement of symptoms and radiological findings [3]. Clinically, aortic vasculitis presents with very nonspecific symptoms such as fever, weight loss, fatigue, abdominal, back, or thoracic pain associated with embolic or ischemic phenomena, and intermittent claudication [4]. Diagnosis is generally established by means of imaging techniques as CT, angio-CT, MRI angiography, PET, and characteristic histopathological findings [5]. The most representative cases in the current literature have described that aortitis is more frequent in men than in women; additionally, most individuals present involvement of the ascending aorta, associated with high levels of C-reactive protein (range from 21.4 to 33.7 mg/dl) and positivity of anti-DNA [6-8]. In some cases, it has been suggested that the pathophysiological mechanisms of serositis and aortitis are shared since they sometimes present together and usually in the absence of major organ involvement (lupus nephritis, skin, or central nervous system involvement), quite the contrary to this case. There are contradictory data regarding the deposition of immune complexes and vascular compromise; despite this, a case of an autopsy is described showing that aortitis was mediated by immune complexes with the deposition of IgG, C3, as well as fibrinogen in the wall of the aorta. This case also presented an associated APS, which could be possibly related to the pathophysiology of vascular compromise, however, the findings of the pathology show a clear vasculitic component, which is explained better by the activity of the disease. Additionally, the majority of reported cases did not show positivity for antiphospholipid antibodies or for the definition of the syndrome [9]. Reports have proposed the use of mycophenolate as maintenance therapy following the start of glucocorticoids, without conclusive results regarding the efficacy and, in the case of catastrophic APS, combined treatment with anticoagulants, immunoglobulin, plasmapheresis, and rituximab was indicated, with good results [10]. Given the profile and characteristics of the patient, treatment with cyclophosphamide was indicated pending evaluation of clinical results.

## Conclusions

Given that aortitis is a rare complication in SLE, it has not been possible to homogeneously characterize the clinical, pathological, and immunological findings in the reported cases that may be involved in the pathophysiology of the disease, nor to give a more adequate approach regarding its treatment. Glucocorticoid therapy is still very controversial, however, intermediate doses can have a good result regarding the improvement of symptoms and radiological findings. Acute phase reactants have been found to be elevated in some reported cases, even suggesting an inflammatory involvement of vascular compromise and proposing the blockade of interleukin 6 (IL-6) as a therapeutic alternative, despite this, the levels of C-reactive protein in this case never reached positive values. It is noteworthy that lupus aortitis can also compromise the abdominal aorta and its visceral branches, changing the paradigm that it only affects the ascending aorta. The positivity of anti-DNAs and the consumption of complement seem to be the main characteristic of cases that manifest aortitis. Furthermore, it is believed that its pathophysiology may be more related to the activity of the disease. It is necessary to have the results of the different treatments required by each of the reported cases in order to propose a better therapeutic approach.
